# Sphingosine 1-phosphate enhances the excitability of rat sensory neurons through activation of sphingosine 1-phosphate receptors 1 and/or 3

**DOI:** 10.1186/s12974-015-0286-8

**Published:** 2015-04-12

**Authors:** Chao Li, Jun-nan Li, Joanne Kays, Miguel Guerrero, Grant D Nicol

**Affiliations:** Medical Neuroscience Program, School of Medicine, Indiana University, Indianapolis, IN 46202 USA; Department of Chemistry, The Scripps Research Institute, La Jolla, CA 92037 USA; Department of Pharmacology and Toxicology, School of Medicine, Indiana University, 635 Barnhill Drive, Indianapolis, IN 46202 USA; Department of Pharmacology, Harbin Medical University, Harbin, Peoples’ Republic of China

**Keywords:** Excitability, Sensitization, Sphingosine 1-phosphate, Sensory neuron, Dorsal root ganglia

## Abstract

**Background:**

Sphingosine-1-phosphate (S1P) is a bioactive sphingolipid that acts through a family of five G-protein-coupled receptors (S1PR_1–5_) and plays a key role in regulating the inflammatory response. Our previous studies demonstrated that rat sensory neurons express the mRNAs for all five S1PRs and that S1P increases neuronal excitability primarily, but not exclusively, through S1PR_1_. This raises the question as to which other S1PRs mediate the enhanced excitability.

**Methods:**

Isolated sensory neurons were treated with either short-interfering RNAs (siRNAs) or a variety of pharmacological agents targeted to S1PR_1_/R_2_/R_3_ to determine the role(s) of these receptors in regulating neuronal excitability. The excitability of isolated sensory neurons was assessed by using whole-cell patch-clamp recording to measure the capacity of these cells to fire action potentials (APs).

**Results:**

After siRNA treatment, exposure to S1P failed to augment the excitability. Pooled siRNA targeted to S1PR_1_ and R_3_ also blocked the enhanced excitability produced by S1P. Consistent with the siRNA results, pretreatment with W146 and CAY10444, selective antagonists for S1PR_1_ and S1PR_3_, respectively, prevented the S1P-induced increase in neuronal excitability. Similarly, S1P failed to augment excitability after pretreatment with either VPC 23019, which is a S1PR_1_ and R_3_ antagonist, or VPC 44116, the phosphonate analog of VPC 23019. Acute exposure (10 to 15 min) to either of the well-established functional antagonists, FTY720 or CYM-5442, produced a significant increase in the excitability. Moreover, after a 1-h pretreatment with FTY720 (an agonist for S1PR_1_/R_3_/R_4_/R_5_), neither SEW2871 (S1PR_1_ selective agonist) nor S1P augmented the excitability. However, after pretreatment with CYM-5442 (selective for S1PR_1_), SEW2871 was ineffective, but S1P increased the excitability of some, but not all, sensory neurons.

**Conclusions:**

These results demonstrate that the enhanced excitability produced by S1P is mediated by activation of S1PR_1_ and/or S1PR_3_.

## Background

Sphingosine-1-phosphate (S1P) is a bioactive lipid which has been shown to exert important biological functions in a variety of systems such as the immune and cardiovascular systems as well as in the regulation of cancer cells [1-4]. S1P can function as a primary messenger to act on a family of five G-protein-coupled receptors (S1P receptors, S1PR_1–5_) (reviewed by [5,6]). Several recent studies also demonstrate that S1P is involved in the sensation and modulation of pain (reviewed by [7,8]). Previous work from our laboratory demonstrated that extracellular delivery of S1P was capable of enhancing the excitability of sensory neurons in a GDP-β-S-dependent manner [9]. Additional studies demonstrated that S1P activation of S1PRs augmented both heat- and capsaicin-activated membrane currents in mouse sensory neurons [10]. Application of S1P increased the firing frequency of polymodal C fibers in response to a thermal stimulus in a skin-nerve preparation, suggesting that this sensitization was not a result of immune cell invasion [10]. Similarly, injection of S1P into the rat’s hindpaw produced edema, which is a hallmark of inflammation [11,12] as well as significant thermal and mechanical hyperalgesia [10,13]. Recent single-cell quantitative real-time PCR studies from our laboratory demonstrated that small-, medium-, and large-diameter sensory neurons can express the mRNAs for all five S1PRs wherein S1PR subtype 1 (S1PR_1_) was the highest expressor in greater than 50% of these isolated single neurons [14].

To establish which S1PR mediated the enhanced excitability produced by S1P, a study using short-interfering RNA (siRNA) to selectively knockdown expression and selective agonists demonstrated that S1PR_1_ plays a crucial, but not exclusive, role in mediating neuronal sensitization. Small-diameter sensory neurons treated with siRNA targeted to S1PR_1_ were unresponsive to the S1PR_1_ selective agonist SEW2871; however, treatment with the more global agonist, S1P, was still capable of increasing the excitability in approximately one third of the siRNA-treated neurons [15]. Thus, these observations indicated that S1PR_1_ plays a prominent role in the S1P-induced neuronal sensitization, but there must be other S1P receptors capable of mediating the S1P-induced enhancement of excitability. The studies described below show that, in addition to S1PR_1_, activation of S1PR_3_ can lead to the enhancement of excitability in sensory neurons.

## Methods

### Isolation and maintenance of sensory neurons

Sensory neurons were harvested from young adult Sprague–Dawley rats (80 to 150 g) and from young adult mice on a C57BL/6 J background (Harlan Laboratories, Indianapolis, IN, USA). Sensory neurons isolated from the mouse were only used in the examination of membrane currents activated by S1P. Briefly, male rats or mice were killed by placing them in a chamber that was then filled with CO_2_. Dorsal root ganglia (DRGs) were isolated and collected in a conical tube with sterilized Puck’s solution. The tube was centrifuged for 1 min at approximately 2000 × *g*, and the pellet was resuspended in 1 ml Puck’s solution containing 10 U of papain (Worthington, Lakewood, NJ, USA). After a 15-min incubation at 37°C, the tube was centrifuged at 2000 × *g* for 1 min, and the supernatant was replaced by 1 ml F-12 medium containing 1 mg collagenase IA and 2.5 mg dispase II (Roche Diagnostics, Indianapolis, IN, USA). The DRGs were resuspended and incubated at 37°C for 20 min. The suspension was centrifuged for 1 min at 2000 × *g*, and the supernatant was removed. The pellet was resuspended in F-12 medium supplemented with 10% heat-inactivated horse serum and 30 ng/ml nerve growth factor (NGF) (Harlan Bioproducts, Indianapolis, IN, USA) and mechanically dissociated with a fire-polished glass pipette until all visible chunks of tissue disappeared. Isolated cells were plated onto plastic coverslips previously coated with 100 μg/ml poly-d-lysine and 5 μg/ml laminin. Cells were maintained in culture at 37°C and 3% CO_2_ for 18 to 24 h before electrophysiological recording. All procedures have been approved by the Animal Use and Care Committee of the Indiana University School of Medicine.

### Electrophysiology

Recordings were made using the whole-cell patch-clamp technique as previously described [16]. Briefly, a coverslip with sensory neurons was placed in a recording chamber filled with normal Ringer’s solution of the following composition (in mM): 140 NaCl, 5 KCl, 2 CaCl_2_, 1 MgCl_2_, 10 4-(2-Hydroxyethyl)piperazine-1-ethanesulfonic acid (HEPES), and 10 glucose, with pH adjusted to 7.4 using NaOH. Recording pipettes were pulled from borosilicate glass tubing (Model G85165T-4, Warner Instruments, Hamden, CT, USA). Recording pipettes had resistances of 2 to 5 MΩ when filled with the following solution (in mM): 140 KCl, 5 MgCl_2_, 4 ATP, 0.3 GTP, 0.25 CaCl_2_, 0.5 EGTA (calculated free Ca^2+^ concentration of 100 nM, MaxChelator), and 10 HEPES, at pH 7.2 adjusted with KOH. Whole-cell voltages or currents were recorded with an Axopatch 200 or Axopatch 200B amplifier (Molecular Devices, Sunnyvale, CA, USA). Data were acquired and analyzed with pCLAMP 10 (Molecular Devices, Sunnyvale, CA, USA). All drugs were applied with a VC-8 bath perfusion system (Warner Instruments, Hamden, CT, USA) unless otherwise noted. In the current-clamp experiments, the neurons were held at their resting potentials (between −45 and −65 mV), and a depolarizing current ramp (1,000 ms in duration) was applied. The amplitude of the ramp was adjusted to produce between 2 and 4 action potentials (APs) under control conditions and then the same ramp was used throughout the recording period for each individual neuron. Voltages were filtered at 5 kHz and sampled at 2 kHz. In voltage-clamp recordings, neurons were held at −60 mV. Currents were filtered at 5 kHz and sampled at 500 Hz. Additionally, the voltage-clamp recordings were digitally filtered after acquisition using a low-pass 8-pole Bessel filter function (60 Hz −3 dB cutoff) in Clampfit. At the end of each recording, the neuron was exposed to 1 μM capsaicin. This neurotoxin was used to distinguish capsaicin-sensitive sensory neurons as these neurons are believed to transmit nociceptive information [17]. However, the correlation between capsaicin sensitivity and that a neuron is a nociceptor is not absolute. Some nociceptive neurons are insensitive to capsaicin and some capsaicin-sensitive neurons are not nociceptors [18]. Therefore, this agent was used to define a population of small-diameter sensory neurons that could serve a nociceptive function. All results presented in this report were obtained from capsaicin-sensitive neurons, unless otherwise stated. All experiments were performed at room temperature, approximately 23°C.

### siRNA treatment

The gene sequences of S1PR_2_ and S1PR_3_ were obtained from NCBI with the accession numbers NM_017192 and XM_225216, respectively. siRNAs targeting S1PR_2_ and S1PR_3_ were designed by the online tool provided by the Whitehead Institute for Biomedical Research, Cambridge, MA (http://sirna.wi.mit.edu) [19] and synthesized by Thermo Scientific (Waltham, MA, USA). Both siRNAs were labeled with the fluorescent tag, fluorescein, with 3′-end modification. For the siRNA targeted to S1PR_2_, the sense strand was 5′-CCUUCUGGUGCUAAUCGCAUU-3′, and the antisense strand was 3′-UUGGAAGACCACGAUUAGCGU-5′. For the siRNA targeted to S1PR_3_, the sense strand was 5′-CAUUCUGAUGUCCGGUAGGUU-3′, and the antisense strand was 3′-UUGUAAGACUACAGGCCAUCC-5′. The siRNA targeted to S1PR_1_ was the same sequence as described in [15] and labeled with the fluorescent tag DY547. A universal Silencer Negative Control #1 siRNA (cat #4390843, Ambion, Grand Island, NY, USA) was used as the negative control.

Neurons isolated from the rat DRG were maintained in culture in F-12 medium with 30 ng/ml NGF at 37°C for 24 h. F-12 was replaced with Opti-MEM medium (Life Technologies, Grand Island, NY, USA), and the neurons were incubated at 37°C for about 5 h for lipid transfection. The transfection reagent, metafectene (Biontex-USA, San Diego, CA, USA) and siRNA complex (5 μl, 100 nM) were prepared in 2 ml Opti-MEM. Neurons were exposed to either siRNA, negative control siRNA, or metafectene alone and maintained at 37°C for 48 h. F-12 medium was used to wash out the metafectene and the siRNA; neurons were then maintained in F-12 medium with 10% heat-inactivated horse serum and 30 ng/ml NGF. Neurons were incubated for an additional 48 h before real-time quantitative PCR (qPCR) or patch-clamp experiments were performed.

### cDNA generation from siRNA-treated cells

Sensory neurons that had undergone siRNA treatments were collected for real-time qPCR measurements. The F-12 medium was aspirated from the cell-culture dish, and neurons were washed with PBS solution. Total RNA from the cells was extracted by using the RNeasy Plus Mini Kit (Qiagen, Valencia, CA, USA), following the manufacturer’s instructions. The concentration of each individual RNA from different treatments was measured with a NanoDrop ND-1000 Spectrophotometer (Thermo Scientific, Waltham, MA, USA). To eliminate contamination by genomic DNA, 500 ng of RNA was treated with 1 μl DNase I (Invitrogen, cat. #18068-015) in a 10-μl reaction at room temperature for 15 min. The reaction was terminated by adding 1 μl 25 mM ethylenedinitrilo-tetraacetic acid (EDTA), and the reaction mixture was incubated at 65°C for 10 min. To generate cDNA from RNA, the DNase-I-treated RNA template was mixed with 1 μl iScript reverse transcriptase in a 20-μl reaction (iScript cDNA Synthesis Kit cat #170-8891, Bio-Rad, Hercules, CA, USA). The reaction protocol was as follows: 25°C for 5 min, 42°C for 30 min, and 85°C for 5 min.

### Pre-amplification of cDNA from siRNA-treated cells

A 0.5X pooled assay mix was prepared by adding 2 μl of 20X TaqMan® Gene Expression Assay for each gene of interest (GOI) to Tris-EDTA (TE) buffer pH 8.0, final volume 80 μl. All Gene Expression Assays are labeled with the reporter dye FAM, except for hypoxanthine phosphoribosyltransferase 1 (HPRT) which was labeled with the reporter dye VIC. To each 1 μl (25 ng) of cDNA, 5 μl of 2X Pre-amp Master Mix (Life Technologies, Grand Island, NY, USA, cat #4391128), 1 μl of 0.5X pooled assay mix, and 3 μl nuclease-free H_2_O (Ambion, cat #9932) were added. After a 10-min incubation at 95°C, 14 cycles of 95°C/15 s and 60°C/4 min were run, followed by storage at −20°C.

### TaqMan quantitative qPCR

The pre-amplified cDNA was diluted fivefold with nuclease-free H_2_O, and 2.5 μl of the dilution was used as the template in a 10-μl qPCR reaction also containing 5 μl 2X Taqman Gene Expression Master Mix (Applied Biosystems, Waltham, MA, USA, cat #4369514), 0.5 μl 20X TaqMan GOI Assay, and 2 μl nuclease-free water. A positive control template was 25 ng of pooled rat lung cDNA. Reactions were run in triplicate on a 7500 Fast Real-Time PCR System (Applied Biosystems, Waltham, MA, USA). The thermal-cycling condition was 95°C for 10 min followed by 40 cycles of 95°C for 15 s and 60°C for 1 min. The quantification cycle (Cq) values of various GOIs were obtained at the threshold where the value of normalized fluorescence emission generated by FAM or VIC (ΔRn) reached 0.3. The expression of different genes was calculated based on the number of copies of each gene where Number of Copies = (Primer Efficiency)^−Cq^. The relative expression of the GOI was determined by dividing the average copy number of the GOI by that of the reference genes, acidic ribosomal phosphoprotein P0 (Arbp) or HPRT. Efficiencies of each primer pair were determined from the slope of a seven-point standard curve (details described in [14]).

### Data analysis

Data are presented as the means ± standard error of the mean (SEM). Statistical differences in the mRNA expression levels between the control groups and the treatment groups were determined by either Student’s *t*-test or an analysis of variance (ANOVA). Statistical differences between the control recordings and those obtained under various treatment conditions were determined by either an ANOVA or a repeated measures (RM) ANOVA whenever appropriate. When a significant difference was obtained with an ANOVA, *post hoc* analyses were performed using a Holm-Sidak all-pairs test. If the data set failed the normality test, a Kruskal-Wallis one-way ANOVA on ranks was performed, followed by a Tukey or Dunn’s all pairwise test. The results were considered statistically significant when the *P* value was <0.05 (SigmaStat 3.5 software).

### Chemicals

F-12 Nutrient Mixture (Gibco Catalog # 21700–075) was supplemented with the following per liter: 1.18 g NaHCO_3_ (Sigma cat # S6014), 1X (2 mM) L-glutamine (Gibco cat # 25030–081), 50 units penicillin-50 mg/ml streptomycin (Gibco cat #15070-063), 10% heat-inactivated horse serum (Gibco cat #26050-088), 9 μg/ml 5-fluoro-2′-deoyuridine (Sigma cat # F-0503), and 21 μg/ml uridine (Sigma cat #U-3750). S1P and VPC 23019 were obtained from Avanti Polar Lipids (Alabaster, AL, USA); S1P was dissolved according to the manufacturer’s instructions (http://www.avantilipids.com/index.php?option=com_content&view=article&id=1114&Itemid=173&catnumber=860492). Prostaglandin E_2_ (PGE_2_), W146, FTY720, sphingosine kinase inhibitor II (SKI-II), SEW2871, and CAY10444 were purchased from Cayman Chemical (Ann Arbor, MI, USA). CYM-5442 was purchased from Tocris Bioscience (Bristol, UK). VPC 44116 was a generous gift from Dr. Kevin R. Lynch, University of Virginia. All other chemicals were obtained from Sigma-Aldrich (St. Louis, MO, USA). PGE_2_, W146, FTY720, SKI-II, SEW2871, CAY10444, VPC 23019, and VPC 44116 were dissolved in 1-methyl-2-pyrrolidinone (MPL). The MPL stock solutions were then diluted with Ringer’s solution to yield the appropriate concentrations. The vehicle, MPL was typically used at 1,000- to 5,000-fold dilutions. Our earlier studies demonstrated that MPL does not affect the potassium or sodium currents in the DRG sensory neurons [9,20].

## Results

### siRNAs effectively and specifically knock down S1PR expression

**Figure 1:**
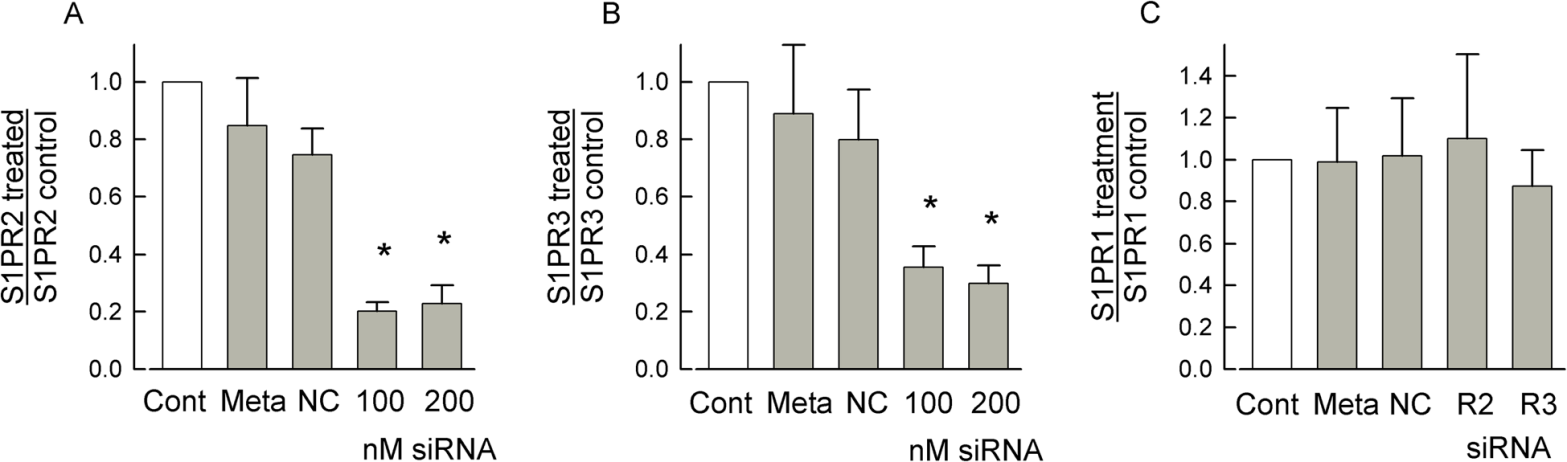
siRNAs targeted to S1PR_2_ or R_3_ specifically knockdown the mRNA levels for their respective receptors. (**A**) 100 and 200 nM siRNA targeted to S1PR_2_ significantly reduced the mRNA levels of S1PR_2_ by 79.8% ± 3.1% and 77.1% ± 6.4%, respectively, compared to the untreated control values. There was no difference between the knockdown values for 100 and 200 nM siRNA treatments. The different treatment groups (Cont - untreated control, Meta - metafectene alone, NC - negative control siRNA, and siRNA targeted to the S1PR) were normalized to their respective untreated control values. (**B**) 100 and 200 nM siRNA targeted to S1PR_3_ significantly reduced the mRNA levels of S1PR_3_ by 64.5% ± 7.3% and 70.2% ± 6.3%, respectively, compared to the untreated control values. There was no difference between the knockdown values for 100 and 200 nM siRNA treatments. (**C**) siRNA targeted to S1PR_2_ (200 nM) or S1PR_3_ (200 nM) does not alter the mRNA levels of S1PR_1_ (*P* = 0.88). (A-C) values were obtained from neurons isolated from five different tissue harvests; a Pfaffl analysis [70] was used to quantify the values of receptor mRNA relative to the reference gene, Arbp, for the different treatments; a Kruskal-Wallis ANOVA with a Tukey *post hoc* test was used to determine statistical differences between the different groups where the asterisks indicate *P* < 0.05. S1PR - sphingosine-1-phosphate receptor, siRNA - short-interfering RNA.

**Figure 2:**
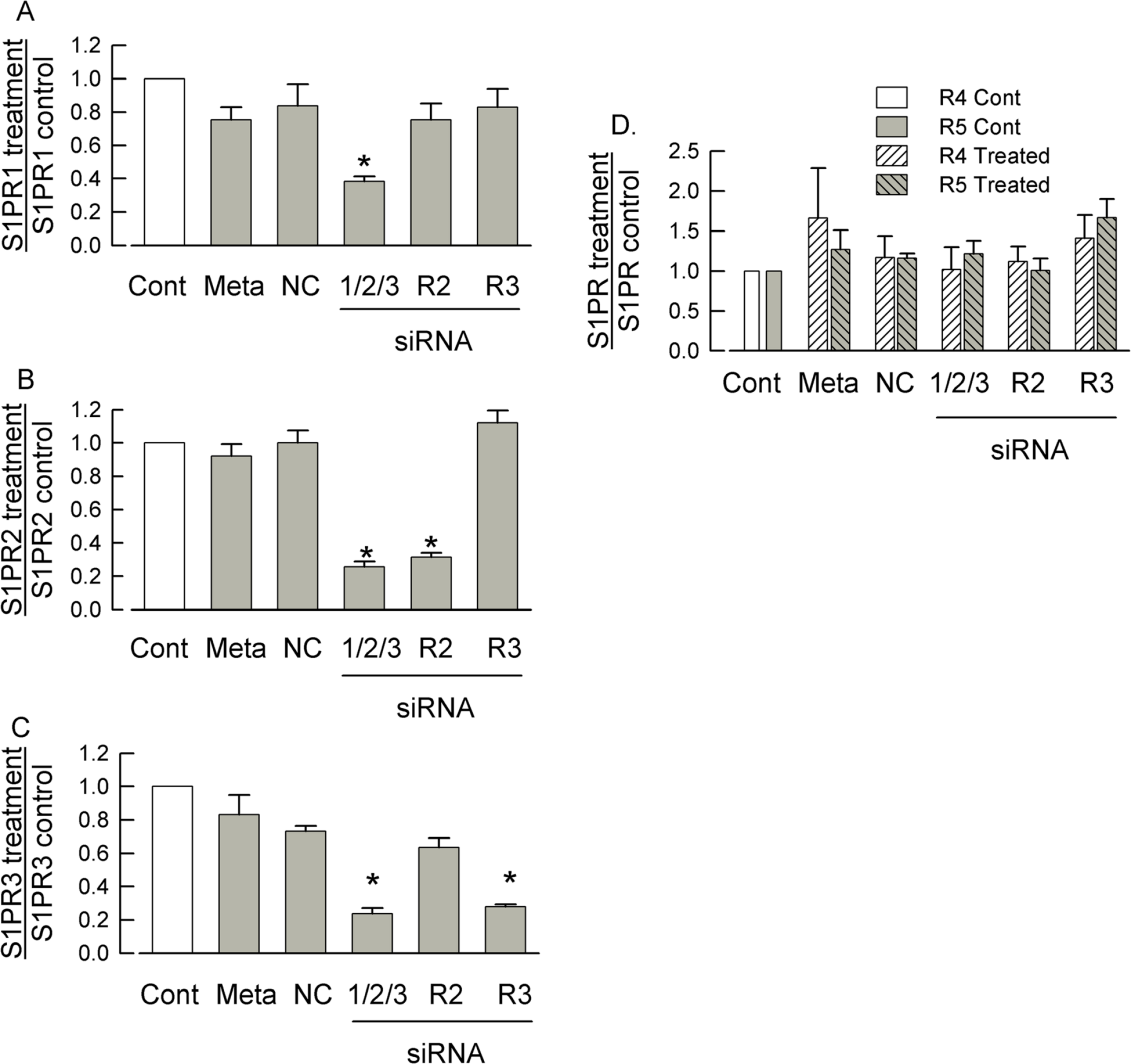
siRNAs targeted to S1PR_1_, R_2_, and R_3_ specifically knockdown receptor expression. (**A**) Combined short-interfering RNAs (siRNAs) targeted to S1PR_1_/R_2_/R_3_ (100 nM each) significantly reduced the mRNA expression of S1PR_1_ by 61.6% ± 2.9% compared to the untreated control (Cont). The levels of S1PR_1_ mRNA were not affected by treatment with metafectene (Meta), 200 nM of the negative control siRNA (NC), 200 nM siRNA targeted to S1PR_2_ (R2), or 200 nM siRNA targeted to S1PR_3_ (R3). The different treatment groups were normalized to their respective untreated controls. (**B**) Combined siRNAs targeted to S1PR_1_/R_2_/R_3_ (100 nM each) and siRNA targeted to S1PR_2_ (200 nM) alone significantly reduced the expression of S1PR_2_ mRNA by 74.3% ± 3.2% and 68.5% ± 2.6%, respectively. (**C**) Combined siRNAs targeted to S1PR_1_/R_2_/R_3_ (100 nM each) and siRNA targeted to S1PR_3_ (200 nM) alone significantly reduced the expression of S1PR_3_ mRNA by 76.3% ± 3.5% and 72.1% ± 1.5%, respectively. (**D**) The combined siRNAs targeted to S1PR_1_/R_2_/R_3_ did not significantly affect the mRNA levels of either S1PR_4_ or R_5_ (*P* = 0.88 and 0.20, respectively, ANOVA). (A-D) Values were obtained from neurons isolated from four different tissue harvests; a copy number analysis (see Kays *et al*. [14]) was used to quantify the values of receptor mRNA relative to the reference gene, Arbp, for the different treatments; a Kruskal-Wallis ANOVA with a Tukey *post hoc* test was used to determine statistical differences between the different groups where the asterisks indicate *P* < 0.05.

Our previous studies demonstrated that S1PR_1_ played a predominate, but not exclusive, role in augmenting the excitability of rat sensory neurons [15]. These results raise the question as to which other S1PRs contribute to the S1P-mediated sensitization. The existing literature indicates that in other model systems as well as in the nervous system S1PR_1_, R_2_, and R_3_ play important although varied roles in modulating cellular function; however, the impact of S1PR_4_ and R_5_ are poorly understood. To explore the idea that S1PR_1_, R_2_, and R_3_ are key players in the S1P-mediated sensitization, siRNA targeted to these S1PRs were designed and their ability to reduce the expression of their respective receptor was measured by qPCR. Our previous results showed that siRNA targeted to S1PR_1_ reduced its expression by about 75% [15]; this siRNA was used in experiments described below. Treatment with siRNAs (100 and 200 nM) targeted to S1PR_2_ or R_3_ significantly reduced the levels of mRNA compared to naïve untreated neurons by approximately 80% and 70%, respectively (see Figure 1A,B). Treatment with the transfecting detergent, metafectene, or the negative control siRNA had no significant effect on the mRNA levels for either S1PR_2_ or R_3_. In addition, siRNA targeted to either S1PR_2_ or R_3_ did not have any off-target effects on the expression levels of S1PR_1_ (see Figure 1C). In order to determine the potential contributions of multiple S1PRs to neuronal sensitization, the siRNAs targeted to S1PR_1_, R_2_, and R_3_ were pooled (100 nM each) to assess their knockdown of the mRNA levels for these individual receptors as well as their possible off-target effects. The combination of S1PR_1_, R_2_, and R_3_ siRNAs reduced the mRNAs for S1PR_1_, R_2_, and R_3_ by 62%, 74%, and 76%, respectively, compared to untreated neurons (see Figure 2A,B,C, respectively) and had no off-target effects. The pooled siRNAs were as equally effective as the individual siRNAs for S1PR_2_ and R_3_ (panels B and C of Figure 2). For example, in Figure 2C, the pooled siRNAs (100 nM each) reduced the mRNA levels of S1PR_3_ by 76%, and the single siRNA to S1PR_3_ (200 nM) reduced S1PR_3_ mRNA by 72%. As shown in Figure 2D, neither the pooled siRNAs nor the individual siRNAs targeted to S1PR_2_ or R_3_ affected the mRNA levels of S1PR_4_ and R_5_. Similar results were obtained when the mRNA levels were assessed relative to the reference gene HPRT (data not shown). Taken together, these results indicate that the siRNAs targeting S1PR_1_, R_2_, or R_3_ specifically reduced the mRNA expression of their respective receptor and have no off-target actions.

### Pooled siRNAs targeted to S1PR_1/2/3_ or S1PR_1/3_ block the S1P-induced increase in excitability

**Figure 3:**
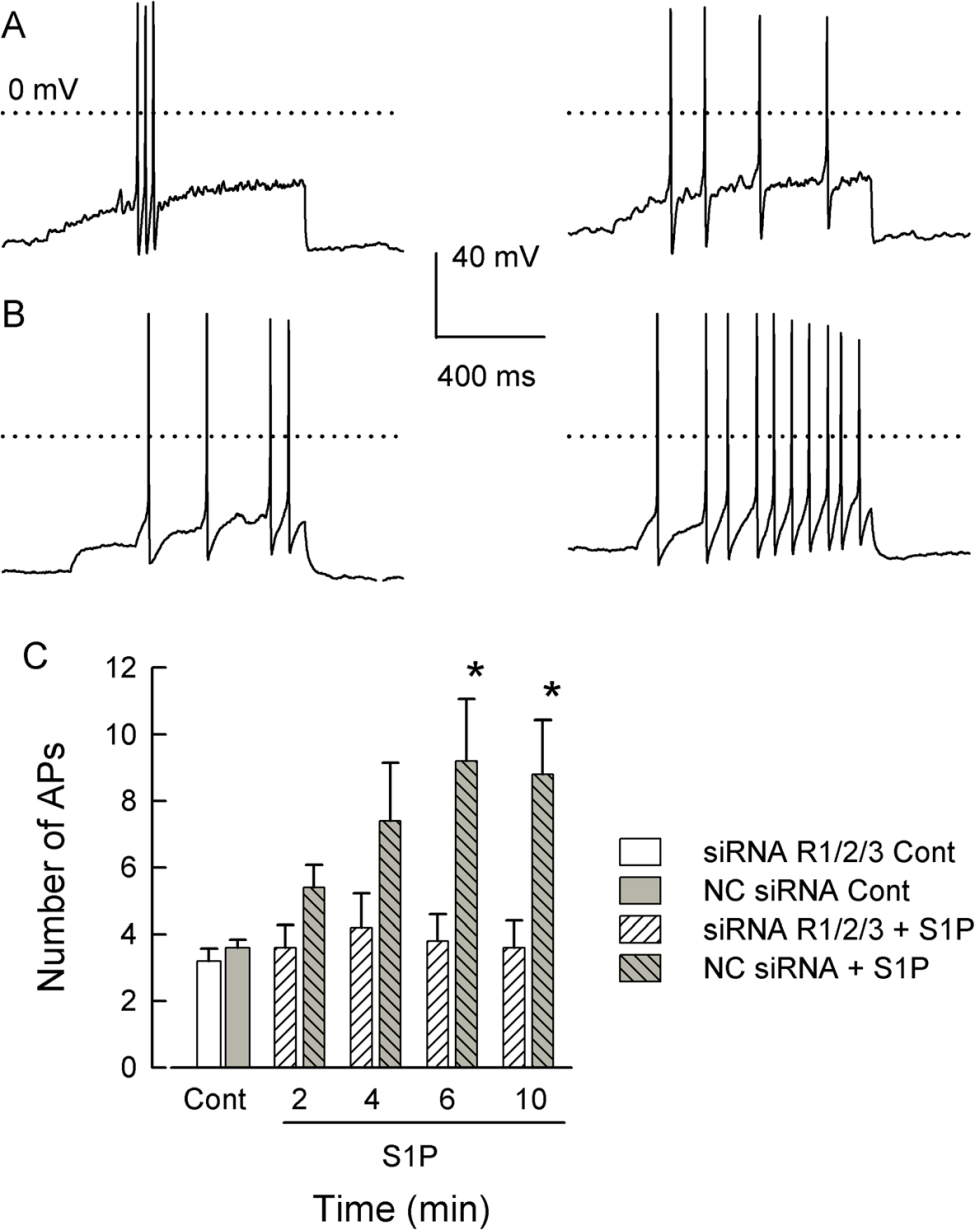
Combined siRNAs targeted to S1PR_1_/R_2_/R_3_ blocked the S1P-induced increase in excitability. (**A**) shows representative traces from a neuron treated with the combined short-interfering RNAs (siRNAs) targeted to sphingosine-1-phosphate (S1P)R_1_/R_2_/R_3_ (100 nM each); the trace shown in the left panel was obtained under control conditions whereas that in the right panel was after a 6-min exposure to 1 μM S1P. The dotted lines indicate the 0 mV level. **(B)** illustrates cells treated with 300 nM negative control siRNA. The left panel shows that under control conditions 4 action potentials (APs) were fired whereas, after a 6-min exposure to 1 μM S1P, the same ramp current elicited 10 APs. (**C**) summarizes the results obtained from five cells in each treatment group. S1P significantly increased the number of APs in neurons treated with negative control (NC) siRNA at 6 and 10 min. The asterisks (*) represent a significant difference compared to the control (*P* < 0.05 RM ANOVA with a Holm-Sidak all-pairs test).

Having validated the specificity of these siRNAs, the functional contributions of S1PRs to the S1P-induced enhancement of neuronal excitability were examined. As shown in the representative traces in Figure 3A, after treatment of sensory neurons with the pool of siRNAs targeted to S1PR_1/2/3_ (100 nM each), the ramp of current evoked only 4 APs after a 6-min exposure to 1 μM S1P (right panel) compared to 3 APs for the control conditions (left panel). In contrast, after treatment with 300 nM negative control (NC) siRNA, a 6-min exposure to 1 μM S1P increased the number of APs from a control value of 4 APs (Figure 3B, left panel) to 10 APs (right panel). The results obtained from a total of five neurons in each treatment group are summarized in Figure 3C. Exposure to 1 μM S1P significantly increased the number of APs in NC siRNA-treated neurons at both 6 and 10 min compared to the control values (*P* = 0.003, RM ANOVA Holm-Sidak all-pairs test) and is similar to our previous reports obtained from untreated sensory neurons [9,15]. However, S1P failed to augment AP firing in those neurons treated with the pooled siRNAs (*P* = 0.47). These results indicate that S1P can sensitize sensory neurons through the activation of S1PR_1_, R_2_, and/or R_3_ and that R_4_ and R_5_ are not sufficient to mediate the S1P-induced sensitization.

**Figure 4:**
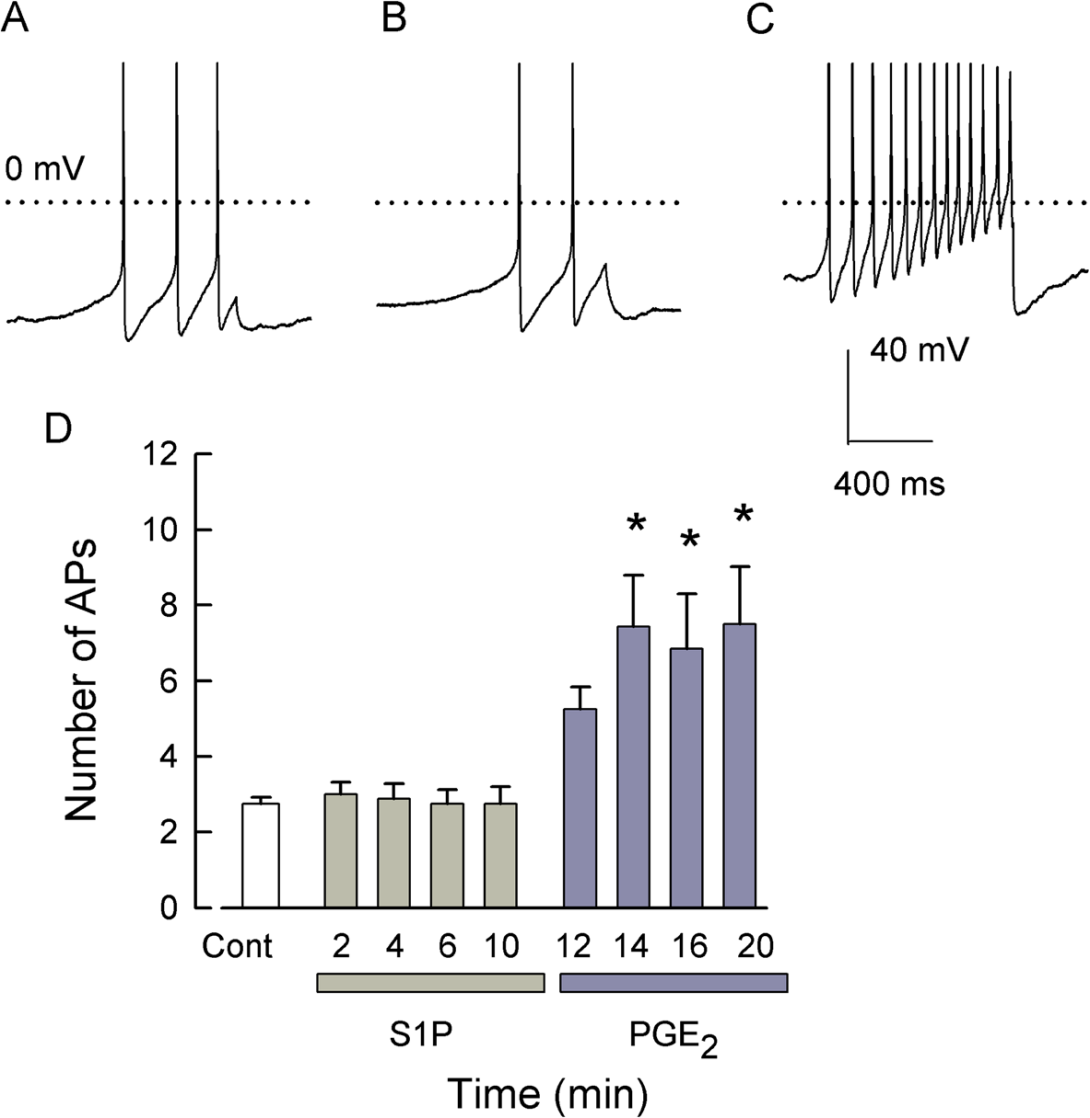
Neurons treated with siRNAs targeted to S1PR_1_ and R_3_ were not sensitized by S1P but did respond to PGE_2_. (**A**) demonstrates representative traces from a sensory neuron treated with combined siRNAs targeted to S1PR_1_ and R_3_ (100 nM each); under control conditions, the neuron generated 3 APs. (**B**) After a 10-min exposure to 1 μM S1P, this neuron fired only 2 APs. (**C**) A subsequent 10-min exposure to 1 μM PGE_2_ resulted in the generation of 13 APs. (**D**) summarizes the effects of S1P and PGE_2_ exposures after treatment with siRNAs targeted to S1PR_1_ and R_3_. S1P failed to augment AP firing, but PGE_2_ significantly increased the number of APs after 14-, 16-, and 20-min exposures. Results were obtained from eight neurons (control through 12 min), seven neurons at 14 min, and six neurons at 16 and 20 min. Asterisks (*) represent a significant difference between those treatments compared to control (*P* < 0.05, Kruskal-Wallis ANOVA on ranks with Dunn’s *post hoc* test). AP - action potential, Cont - control, PGE_2_ - prostaglandin E_2_, S1P - sphingosine-1-phosphate.

Previously, we demonstrated that S1PR_1_ plays a prominent, but not exclusive, role in the sensitization mediated by S1P [15]. In addition, to examine the role of S1PR_2_, we used a putative S1PR_2_-specific antagonist JTE-013; surprisingly, JTE-013 itself increased the excitability of sensory neurons through an as-yet-to-be-defined G-protein-coupled receptor (GPCR) [21]. Thus, to distinguish a possible role for S1PR_2_ in the S1P-induced sensitization, sensory neurons were treated with a combination of siRNAs targeted to S1PR_1_ and R_3_ (100 nM each). As shown in Figure 4, under control conditions, a representative neuron generated 3 APs in response to the current ramp (Figure 4A), and after a 10-min exposure to 1 μM S1P, the ramp evoked 2 APs (Figure 4B). As a positive control, neurons were exposed to pro-inflammatory PGE_2_ to confirm that these neurons were capable of sensitization (via a Gs/cAMP/PKA pathway) [22-24]. After a 10-min exposure to 1 μM PGE_2_, the ramp evoked 13 APs (Figure 4C). As summarized in Figure 4D, S1P failed to enhance the excitability after treatment with siRNAs targeted to S1PR_1_ and R_3_, suggesting that S1PR_1_ and/or R_3_, but not R_2_, mediates the sensitization produced by S1P. In contrast, PGE_2_ significantly increased the AP firing at 14, 16, and 20 min compared to both the control and S1P treatment conditions (*P* < 0.001, ANOVA Holm-Sidak all-pairs test). Taken together, these results demonstrate that the S1P-induced sensitization is mediated through S1PR_1_ and/or R_3_ while R_2_, R_4_, and R_5_ appear to have no significant role.

### The selective S1PR_3_ agonist, CYM-5541, sensitizes sensory neurons

**Figure 5:**
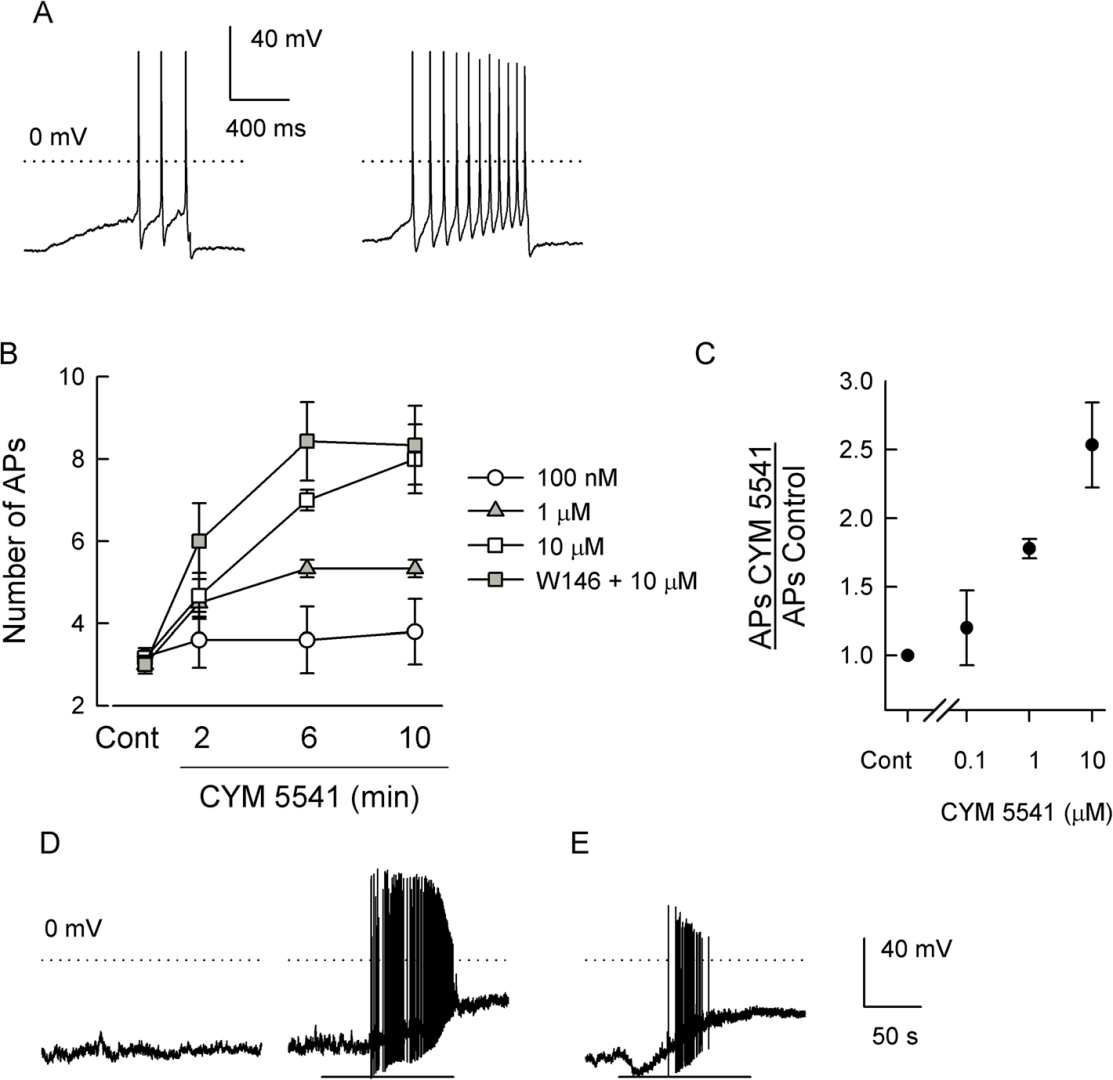
A selective agonist of S1PR_3_, CYM-5541, sensitizes AP firing. (**A**) Left panel illustrates a representative recording where the ramp evoked 3 APs under control conditions; right panel shows that after a 10-min exposure to 10 μM CYM-5441, 11 APs were generated. The dotted line indicates 0 mV. (**B**) the time- and concentration-dependence of CYM-5541 on the number of evoked APs over a 10-min recording period. For 100 nM, there was no effect on the number of evoked APs (*n* = 5, *P* = 0.80 RM ANOVA); for 1 μM, the increase in AP number at 6 and 10 min was significantly different than the control (*n* = 6, *P* = 0.002 RM ANOVA Friedman test on ranks); for 10 μM, the increase at 6 and 10 min was significantly different than the control (*n* = 6, *P* < 0.001 Kruskal-Wallis ANOVA on ranks Dunn’s test); for the 1 μM W146 + 10 μM CYM-5541, the increase at 6 and 10 min was significantly different than the control (*n* = 7 for control, 2 and 6 min, *n* = 6 for 10 min, *P* < 0.001 ANOVA Holm-Sidak all-pairs test). The AP values for 10 μM CYM-5541 and 1 μM W146 + 10 μM CYM-5541 at either 6 or 10 min were not different (*P* > 0.05 ANOVA). (**C**) Concentration dependence for the normalized fold increase in the number of APs measured after a 10-min exposure to CYM-5541. (**D**) Recording of membrane potential where the left panel shows the control condition; the right panel shows exposure to 30 μM CYM-5541 (30 to 150 s represented by the bar). (**E**) Depolarization produced by 30 μM SEW2871 (application 30 to 150 s). Recordings were acquired at 5 kHz; traces are reproduced at 0.5 kHz. Scale bars apply to all three panels. AP - action potential, Cont - control.

Our results suggest that S1PR_3_ can lead to the sensitization of sensory neurons. To test that idea directly, the recently discovered selective agonist of S1PR_3_, CYM-5541, was used [25]. In Chinese hamster ovary cells (CHOs) stably expressing S1PRs, the half-maximal effective concentration (EC_50_) values for CYM-5541 activation of S1PR_3_ was 105 nM and for S1PR_1_ it was approximately 33 μM, and there was no activity at S1PR_2/4/5_ for concentrations as high as 50 μM [25]. We found that CYM-5541 in a time- and concentration-dependent manner lead to the sensitization of AP firing in small-diameter sensory neurons. A representative recording (Figure 5A) shows that, under control conditions, the depolarizing ramp evoked 3 APs whereas, after a 10 min exposure to 10 μM CYM-5441, 11 APs were generated. The time- and concentration-dependence for the actions of CYM-5441 are summarized in Figure 5B. Exposure to 100 nM CYM-5541 failed to alter AP firing (*n* = 5, *P* = 0.80 RM ANOVA) or the resting membrane potential (see Table 1) over a 10-min recording period. Both 1 and 10 μM CYM-5541 significantly enhanced AP firing after 6- and 10-min exposures compared to their respective controls. However, neither of these concentrations of CYM-5541 depolarized the resting membrane potential (see Table 1). In addition, neither the resting membrane potential (−58.5 ± 1.7 mV control *vs*. −57.9 ± 3.0 mV CYM-5541 after 10 min, *n* = 7, *P* = 0.89 ANOVA, data not shown) nor the enhanced excitability produced by 10 μM CYM-5541 were affected by a 30-min pretreatment with 1 μM W146, a S1PR_1_ selective antagonist (inhibition constant (K_i_) 70 to 80 nM) [26] (see Figure 5B). These results demonstrate that at 10 μM, CYM-5541 was capable of augmenting AP firing without changing the resting membrane potential through the activation of S1PR_3_. Figure 5C summarizes the concentration relation for the fold increase in APs generated at 10 min normalized to the number of APs obtained for their respective untreated control recordings; these results show that 10 μM CYM-5541 produces about a 2.5-fold increase in the number of evoked APs through the activation of S1PR_3_. To determine the maximal response, neurons were then exposed to 30 μM CYM-5541; surprisingly, this led to a rapid and large depolarization that was accompanied by a large number of APs (see Figure 5D). The left panel illustrates a 200-s recording of the resting membrane potential under normal control conditions (−51 mV) wherein there is a complete lack of any spontaneous AP activity; the right panel shows that, in this neuron, exposure to 30 μM CYM-5541 (duration 30 to 150 s) depolarized the membrane potential to −23 mV with a significant generation of spontaneous APs. In nine neurons, 30 μM CYM-5541 lead to an average depolarization of approximately 37 ± 3 mV (see Table 1). In four neurons, the recovery from the CYM-5541-induced depolarization was examined. After a 20-min washout with normal Ringers, the membrane potential had recovered by 40% ± 17% (range 4% to 82%). It is possible that this high concentration of CYM-5541 depolarizes the neuronal membrane through activation of S1PR_1_ rather than R_3_ as the EC_50_ for CYM-5541 at S1PR_1_ is approximately 33 μM [25]. To test this idea, sensory neurons were pretreated for 30 min with either 1 or 10 μM W146. In the presence of W146, 30 μM CYM-5541 did not significantly depolarize the resting membrane potential (see Table 1). In support of CYM-5541 activation of S1PR_1_, exposure to 30 μM SEW2871 (a selective S1PR_1_ agonist) produced a significant depolarization (control −56.8 ± 1.2 mV *vs*. −33.0 ± 4.8 mV after SEW2871, *n* = 5, *P* = 0.007 paired *t*-test, data not shown) that was associated with a large increase in spontaneous AP firing (see Figure 5E). These results are similar to those obtained with CYM-5541. Therefore, these results demonstrate that activation of S1PR_3_ can augment AP firing without directly altering the resting membrane potential; however, at the higher and likely unphysiological concentrations, activation of S1PR_1_ by either SEW2871 or CYM-5441 can produce a large depolarization accompanied by extensive AP firing.

**Table 1:** **Effects of CYM-5541 on membrane potential**

**CYM-5541 concentration**	**Untreated control (mV)**	**Posttreatment (mV)**	***n***
100 nM	−55.5 ± 2.4	−52.8 ± 1.9	5
1 μM	−53.9 ± 1.0	−50.5 ± 1.8	6
10 μM	−51.6 ± 1.0	−48.1 ± 1.8	6
30 μM	−55.5 ± 1.8	−18.6 ± 2.9*	9
1 μM W146 + 30 μM	−61.7 ± 2.5	−52.0 ± 5.6	4
10 μM W146 + 30 μM	−52.9 ± 1.2	−47.8 ± 4.0	3

*P < 0.05 paired *t*-test; *n* = number of neurons.

### Together, selective antagonists to S1PR_1_ and R_3_ abolish the S1P-induced sensitization

To corroborate the siRNA findings, specific antagonists were used to block receptor function: W146, a selective S1PR_1_ antagonist, and CAY10444 (BML-241), a selective S1PR_3_ antagonist. Although most studies indicate that CAY10444 is a low-potency antagonist of S1PR_3_ [27-29], a few studies have suggested that this compound lacks specificity [30-32]. CAY1044 (50 μM) has been reported to completely block the S1P-induced increase in intracellular Ca^2+^ in keratinocytes [28]; consistent with this finding, VPC 23019 (described below) also blocked this increase in Ca^2+^. Using a GPCR-β arrestin assay, CAY10444 suppressed the activation of S1PR_3_ with an IC_50_ value of approximately 5 μM [29]. In MCF-7 Neo cells, treatment with S1P produced a significant increase in the phosphorylation of ERK1/2; this increase was greatly suppressed by similar extents after exposure to either 10 μM CAY1044 or siRNA knockdown of S1PR_3_ [33]. In addition, S1P produced a relaxation of contracted coronary artery, which was significantly attenuated by treatment with 10 μM CAY10444 but was unaffected by W146 [34]. In contrast to the above, Jongsma *et al*. reported that in Flp-In-CHO cells, S1P increased intracellular Ca^2+^ and that treatment with 10 μM CAY10444 produced a rightward shift of about tenfold in the EC_50_ value for the mobilization of Ca^2+^ [30]. However, the EC_50_ values for the increases in intracellular Ca^2+^ produced by ATP activation of P2 receptors and phenylephrine activation of α1-adenoreceptors were also right-shifted by 10 μM CAY10444, although the shifts were smaller than that for S1P. Also, CAY10444 did not affect the S1P-mediated decrease in forskolin-elevated levels of cyclic AMP, suggesting that CAY10444 lacked specificity for S1PR_3_. The differences in these results have yet to be resolved.

**Figure 6:**
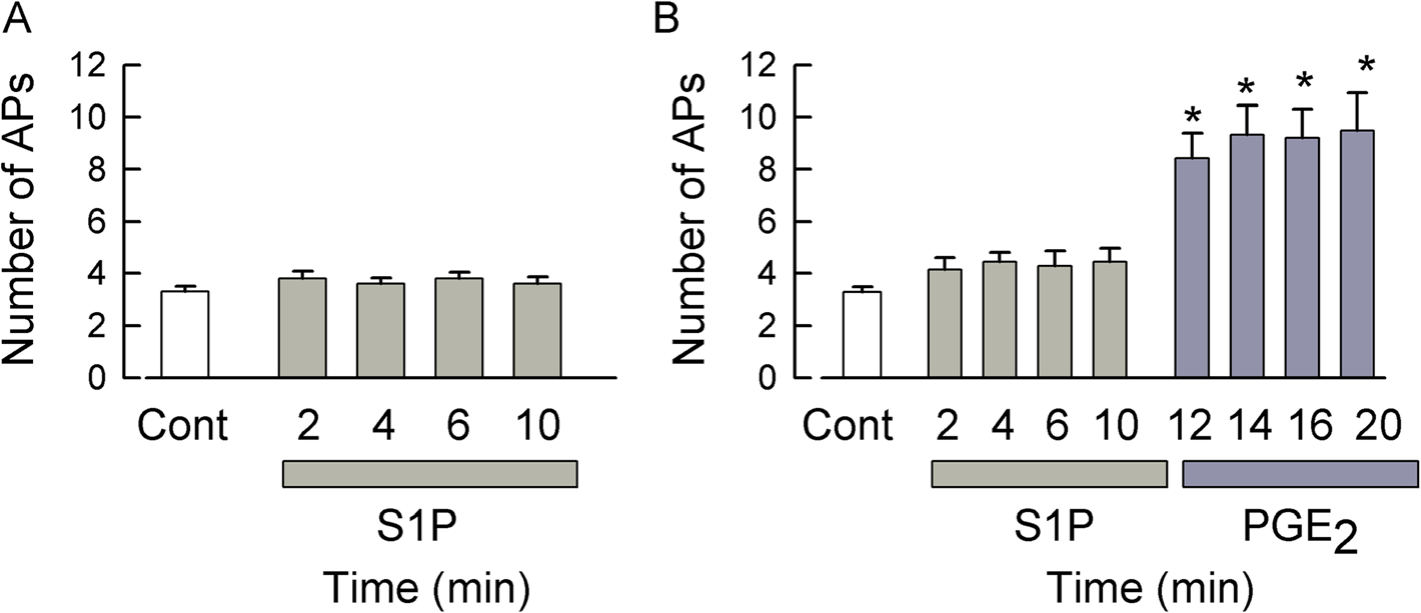
W146, a selective S1PR_1_ antagonist, and CAY10444, a selective S1PR_3_ antagonist, together abolished the sensitizing effect of S1P, but not PGEx_2_. (**A**) Pretreatment with 1 μM W146 and 10 μM CAY10444 for 30 min blocked the sensitization produced by 1 μM S1P. Results were obtained from ten sensory neurons (*P* = 0.15 Friedman RM ANOVA on ranks). (**B**) In another series of experiments, pretreatment with W146 and CAY10444 for 30 min blocked the sensitization produced by 1 μM S1P; however, subsequent exposure to 1 μM PGE_2_ significantly increased the number of evoked APs (*n* = 7 control through 12 min, *n* = 6, 5, and 4 for 14, 16, and 20 min, respectively). The asterisks (*) represent a statistical difference compared to the 10-min S1P results (*P* < 0.05 Kruskal-Wallis ANOVA on ranks followed by Dunn’s *post hoc* test). The 10-min S1P results were not different from the control values. AP - action potential, Cont - control, PGE_2_ - prostaglandin E_2_, S1P - sphingosine-1-phosphate.

To examine the role of S1PR_1_ and S1PR_3_, we found that a 30-min pretreatment with 1 μM W146 and 10 μM CAY10444 blocked the increase in the number of APs after exposure to 1 μM S1P (see Figure 6A, *n* = 10). In a separate series of experiments, treatment with W146 and CAY1044 did not alter the capacity of 1 μM PGE_2_ to significantly increase AP firing (see Figure 6B, *n* = 4 to 7). These results are consistent with our observations obtained with siRNAs targeted to S1PR_1_ and R_3_ and indicate that S1PR_1_ and/or R_3_, but not S1PR_2_, R_4_ or R_5_, is necessary for S1P-induced sensitization.

### VPC 23019 and VPC 44116, antagonists at both S1PR_1_ and R_3_, block S1P-induced sensitization

**Figure 7:**
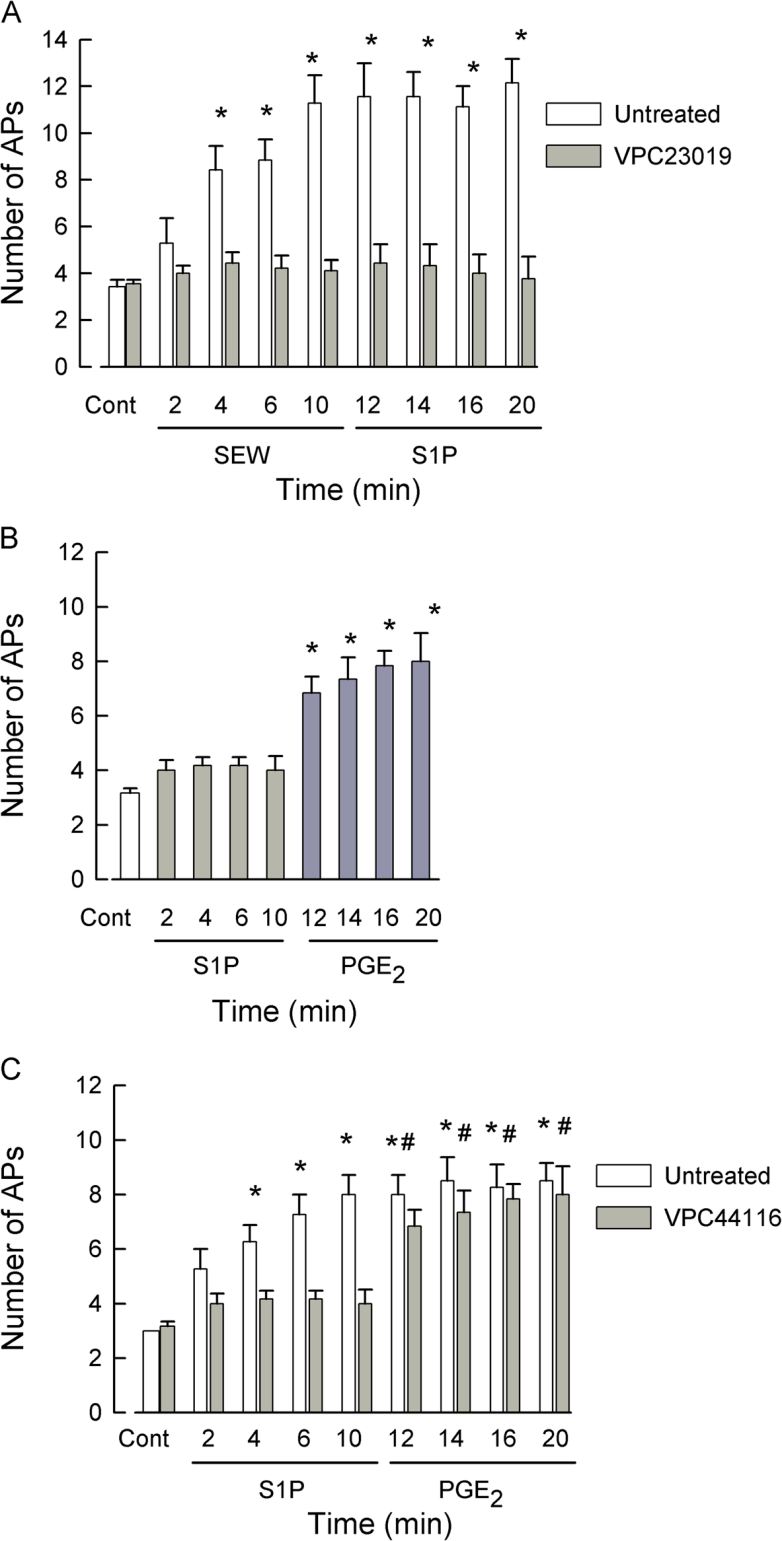
VPC 23019 and VPC 44116, S1PR_1_/R_3_ antagonists and S1PR_4_/R_5_ agonists, block S1P-induced sensitization. (**A**) A 30-min pretreatment with 1 μM VPC 23019 blocked the capacity of 100 nM SEW2871 (SEW), a selective sphingosine-1-phosphate (S1P)R_1_ agonist, and 1 μM S1P, the more global agonist, to augment action potential (AP) firing in sensory neurons (*P* = 0.87, RM ANOVA). In contrast, in untreated neurons, SEW2871 significantly increased the number of evoked APs after only a 4-min exposure (*n* = 7, *P* < 0.001, Friedman RM ANOVA with Tukey *post hoc* test) although the number of evoked APs after S1P was not different from that with SEW2871. (**B**) In a different set of experiments, a 30-min pretreatment with 1 μM VPC 23019 blocked the sensitization produced by 1 μM S1P; however, exposure to 1 μM PGE_2_ significantly increased the AP firing (*P* < 0.05 compared to control, ANOVA followed by Holm-Sidak all-pairs test). (**C**) A 30-min pretreatment with 1 μM VPC 44116 blocked the sensitization produced by 1 μM S1P; however, subsequent expose to 1 μM PGE_2_ significantly increased the number of evoked APs compared to control (represented by asterisks (*)) as well as the number of APs measured at 10-min S1P (represented by #)(*n* = 5, *P* < 0.001, RM ANOVA with Holm-Sidak all-pairs test). Cont - control.

The S1PR_1_/R_3_ antagonist, VPC 23019, was used to further examine the role of S1PR_1_ and R_3_ in the S1P-induced sensitization. VPC 23019 has K_i_ values of approximately 25 and 300 nM for S1PR_1_ and R_3_, respectively, and is also a partial agonist for S1PR_4_ and R_5_ (EC_50_ values of 120 and 480 nM, respectively) [35-37]. Control recordings indicated that exposure to 1 μM VPC 23019 did not change the number of APs in sensory neurons over a 15-min recording period (control 3.8 ± 0.3 APs *vs*. 15-min VPC 23019 4.3 ± 0.9 APs, *n* = 4, *P* = 0.59 RM ANOVA, data not shown). As shown in Figure 7A, a 30-min pretreatment with 1 μM VPC 23019 completely blocked the sensitizing actions of 100 nM SEW2871 (a selective S1PR_1_ agonist) and 1 μM S1P (the more global receptor agonist). In a separate group of sensory neurons, pretreatment with 1 μM VPC 23019 suppressed the enhanced excitability produced by 1 μM S1P but had no effect on the sensitization produced by 1 μM PGE_2_ (see Figure 7B). To corroborate the role of S1PR_1_ and R_3_ in augmenting neuronal excitability, the phosphonate analog of VPC 23019, VPC 44116, was also used. VPC 44116 is a potent antagonist at both S1PR_1_ and R_3_ (K_i_ values of 30 and 300 nM, respectively) and also a partial agonist for S1PR_4_ and R_5_ (EC_50_ values of 6100 and 33 nM, respectively) [36]. Neither VPC 23019 nor VPC 44116 have any effects at S1PR_2_ [32-34]. Similar to VPC 23019, exposure to 1 μM VPC 44116 did not alter the number of evoked APs over a 15-min recording period (control 3.0 ± 0.4 APs *vs*. 15-min VPC 44116 3.8 ± 0.6 APs, *n* = 4, *P* = 0.28 RM ANOVA, data not shown). A 30-min pretreatment with 1 μM VPC 44116 blocked the increase in excitability caused by 1 μM S1P but had no effect on the sensitization produced by 1 μM PGE_2_ (see Figure 7C). Thus, these results demonstrate that the enhanced excitability produced by S1P is mediated by activation of S1PR_1_ and/or R_3_ and that S1PR_2_, R_4_, or R_5_ do not contribute to the enhanced excitability produced by S1P in sensory neurons.

### FTY720, a functional antagonist at S1PR_1/3/4/5_, acutely increases excitability, but prolonged exposure blocks the S1P-mediated sensitization

FTY720 (fingolimod) is a structural analog of sphingosine that upon phosphorylation by sphingosine kinase 2 [38-42] has high affinities for all S1PRs except S1PR_2_ [38,43,44]. Receptor binding and functional assays indicated that FTY720-P has EC_50_ values of approximately 0.3 to 0.6 nM for S1PR_1_, R_4_, and R_5_ and approximately 3 nM for R_3_ but has no activity at S1PR_2_. Thus, FTY720 can act as a potent agonist for specific S1PRs. However, additional studies demonstrated that prolonged incubation with FTY720 resulted in the internalization and degradation of both S1PR_1_ [45-49] and R_3_ [50-53], which in effect removes these receptors from further activation/signaling (but see [49]). Therefore, such pharmacological agents capable of receptor activation with their consequent internalization and degradation have been termed functional antagonists. In this capacity, FTY720-P acts as a suppressor of neuroinflammation and has been approved for the treatment of relapsing-remitting multiple sclerosis [44,54,55]. To further explore the roles of S1PRs in neuronal sensitization, the capacity of FTY720 to act as a functional antagonist was utilized.

**Figure 8:**
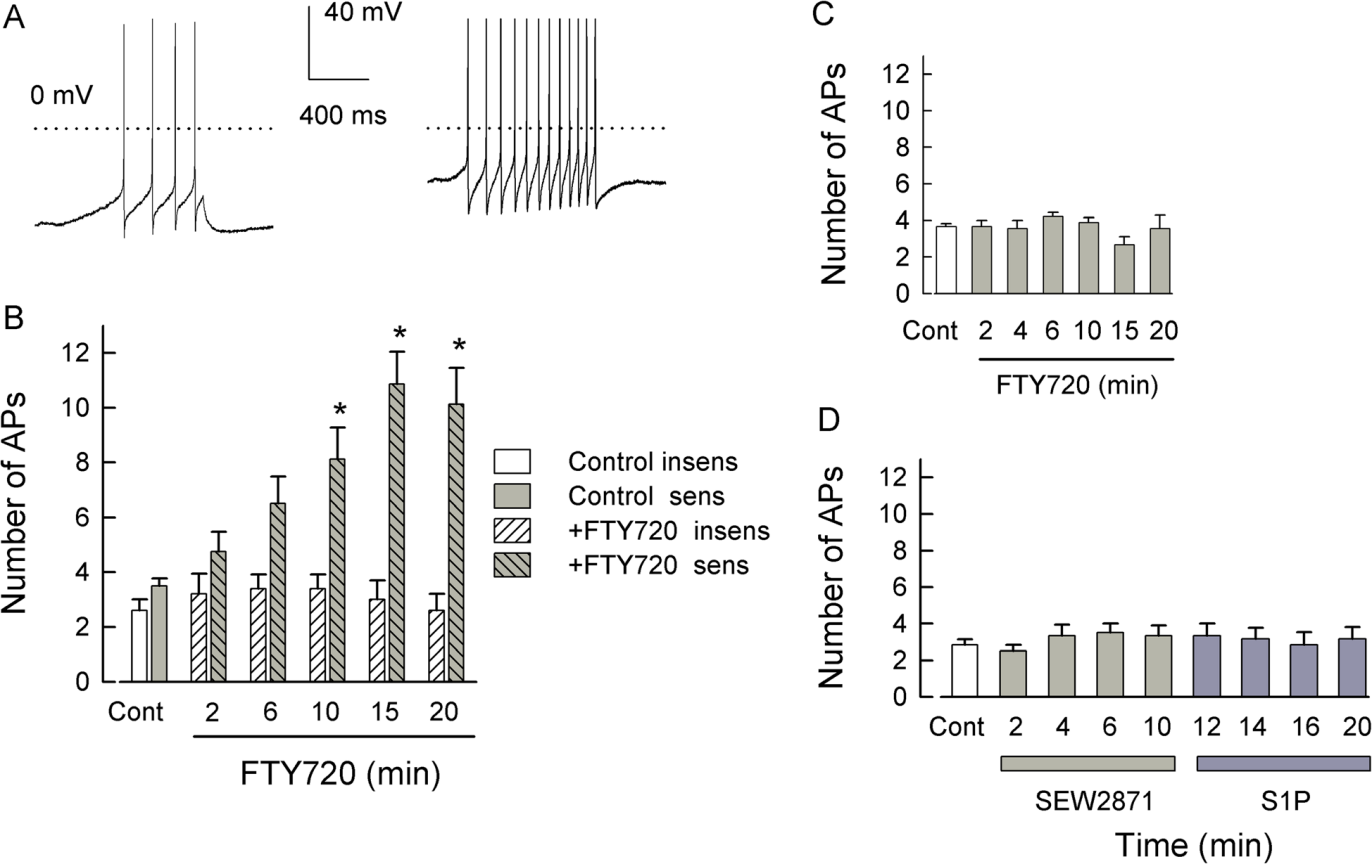
Acute FTY720 exposure increases neuronal excitability in a concentration- and time-dependent manner, but prolonged treatment blocks sensitization. (**A**) shows a representative recording wherein 4 APs were evoked under control conditions (left) whereas, after a 15-min exposure to 100 nM FTY720, the number of evoked APs were increased to 12. The dotted lines indicate the 0 mV level. (**B**) summarizes the effects of 100 nM FTY720 on sensory neurons. In one population, FTY720 significantly increased the number of APs after a 10-min exposure (*n* = 8), whereas another population appeared to be insensitive to FTY720 (*n* = 5). (**C**) A 30-min pretreatment with 5 μM SKI-II, a selective inhibitor of sphingosine kinases, blocked the capacity of 100 nM FTY720 to sensitize sensory neurons (*n* = 9 for the Cont-15 min and *n* = 7 for the 20-min time point, *P* = 0.16 Kruskal-Wallis ANOVA). (**D**) A 1-h pretreatment with 1 μM FTY720 blocks the ability of 100 nM SEW2871 and subsequent 1 μM S1P to sensitize sensory neurons (*n* = 6, *P* = 0.39 Friedman RM ANOVA on ranks). AP - action potential, Cont - control, S1P - sphingosine-1-phosphate.

As expected of a functional antagonist, acute treatment with 100 nM FTY720 produced a significant increase in the excitability of sensory neurons. A representative recording is shown in Figure 8A where 4 APs were evoked by the current ramp under control conditions (left panel); however, after a 15-min exposure to 100 nM FTY720, the ramp now evoked 12 APs (right panel) and depolarized the resting membrane potential from −52 to −28 mV. Similar to our previous findings obtained for SEW2871 [15], a selective S1PR_1_ agonist, sensory neurons were either sensitive or insensitive to FTY720; these results are summarized in Figure 8B. In recordings from 13 neurons, 8 were significantly sensitized after exposure to 100 nM FTY720, exhibiting about a threefold increase in the number of evoked APs after 15- and 20-min exposures. Associated with the increased AP firing, the resting membrane potential was also significantly depolarized from a control value of −54.2 ± 1.2 to −39.3 ± 4.3 mV after a 15-min exposure (data not shown, *P* < 0.001 RM ANOVA, Holm-Sidak all-pairs test). With FTY720-P, the time to reach a significant increase in the number of APs was 15 min which is in contrast to SEW2871 or S1P wherein the time to reach a significant increase in the number of APs typically occurred between 4 and 6 min [9,15]. This delay may well reflect the fact that FTY720 must be phosphorylated by sphingosine kinase 2 and then transported extracellularly (see [56]) where it can function as an agonist at S1PRs (but not S1PR_2_). In contrast, 5 of the 13 neurons were insensitive to FTY720 wherein the number of APs evoked after a 15-min exposure was not different than the control values (control 2.6 ± 0.4 APs *vs*. 15-min exposure 3.0 ± 0.7 APs, *P* = 0.80 Kruskal-Wallis ANOVA). In these FTY720-insensitive neurons, the resting membrane potential was not changed after exposure to FTY720 (data not shown, control −58.8 ± 4.4 mV *vs*. 15-min exposure −58.5 ± 4.6 mV, *P* = 0.98 Kruskal-Wallis ANOVA). Exposure to either 10 or 30 nM FTY720 failed to increase the number of evoked APs in sensory neurons during a 20-min recording period. For example, under control conditions 3.6 ± 0.2 APs were evoked by the current ramp, and after a 20-min exposure to 30 nM FTY720, 4.4 ± 0.5 APs were generated (data not shown, *n* = 5, *P* = 0.92 RM ANOVA). Previous studies indicated that the actions of FTY720 were dependent upon phosphorylation by sphingosine kinase 2 [38-42]. To confirm that phosphorylation was required for the sensitizing actions of FTY720, sensory neurons were pretreated for 30 min with 5 μM SKI-II, a specific inhibitor of sphingosine kinases [57-59], and then exposed to 100 nM FTY720. Inhibition of sphingosine kinases completely blocked the ability of FTY720 to sensitize sensory neurons (see Figure 8C). This result demonstrates that the FTY720-induced increase in neuronal excitability depends on the activity of sphingosine kinases.

The above results demonstrate that FTY720-P acutely augments the excitability of sensory neurons; this finding raises the question as to whether prolonged treatment with this agonist can result in the internalization/degradation of S1PRs (except S1PR_2_) as expected of a functional antagonist. Sensory neurons were pretreated with 1 μM FTY720 for 1 h. As shown in Figure 8D, under control conditions, the ramp evoked an average of 2.8 ± 0.3 APs (*n* = 6). To specifically test whether the sensitization was mediated by activation of S1PR_1_, these sensory neurons were exposed to 100 nM SEW2871, a selective S1PR_1_ agonist. After prolonged treatment with FTY720, SEW2871 failed to augment AP firing (3.3 ± 0.6 APs after a 10-min exposure, *n* = 6). These same neurons were then exposed to the more global agonist, S1P (1 μM), which also did not enhance AP firing (3.2 ± 0.7 APs after a 10-min exposure). There was no significant difference between the control values and any of the treatment time points (*P* = 0.39, RM ANOVA on ranks). Similarly, after pretreatment with FTY720, neither SEW2871 nor S1P had any effect on the resting membrane potential (control −61.8 ± 4.9 mV, 10-min SEW2871 − 63.2 ± 6.4 mV, 10-min S1P −60.6 ± 6.6 mV, *n* = 6, *P* = 0.96 RM ANOVA). In contrast, two untreated sensory neurons isolated from the same tissue harvests were sensitized after exposures to SEW2871 and S1P (control both cells evoked 3 APs, 10-min SEW2871 13 and 8 APs, and 10-min S1P 8 and 10 APs, respectively, data not shown). A 1-h pretreatment with 30 nM FTY720 did not block the capacity of 1 μM S1P to sensitize sensory neurons; in these two neurons, 3 APs were evoked under control conditions whereas, after a 10-min exposure to 1 μM S1P, the ramp evoked 14 and 9 APs. In a separate series of experiments, a 1-h pretreatment with 1 μM FTY720 blocked the capacity of 1 μM S1P to sensitize sensory neurons (data not shown; control 3.6 ± 0.3 APs *vs*. S1P at 10 min 5.0 ± 0.7 APs, *n* = 7); however, this FTY720 pretreatment had no effect on the subsequent enhancement of excitability produced by the exposure to 1 μM PGE_2_ (after 2 min 7.4 ± 0.7 APs and after 10 min 8.2 ± 0.7 APs, *P* < 0.05 *vs*. the control or the 10-min S1P, ANOVA on ranks). Therefore, these results demonstrate that FTY720-P acutely functions as an agonist to increase neuronal excitability and that prolonged treatment with this agonist leads to suppression of sensitization produced by either SEW2871 or S1P. A corollary to this result is that because FTY720-P does not affect S1PR_2_, and S1P failed to enhance the excitability after FTY720 treatment, this would clearly indicate that activation of S1PR_2_ is not sufficient to sensitize sensory neurons.

### Pretreatment with the S1PR_1_ agonist, CYM-5442, prevents SEW2871, but not S1P, from increasing excitability; CYM-5442 also serves as a functional antagonist

**Figure 9:**
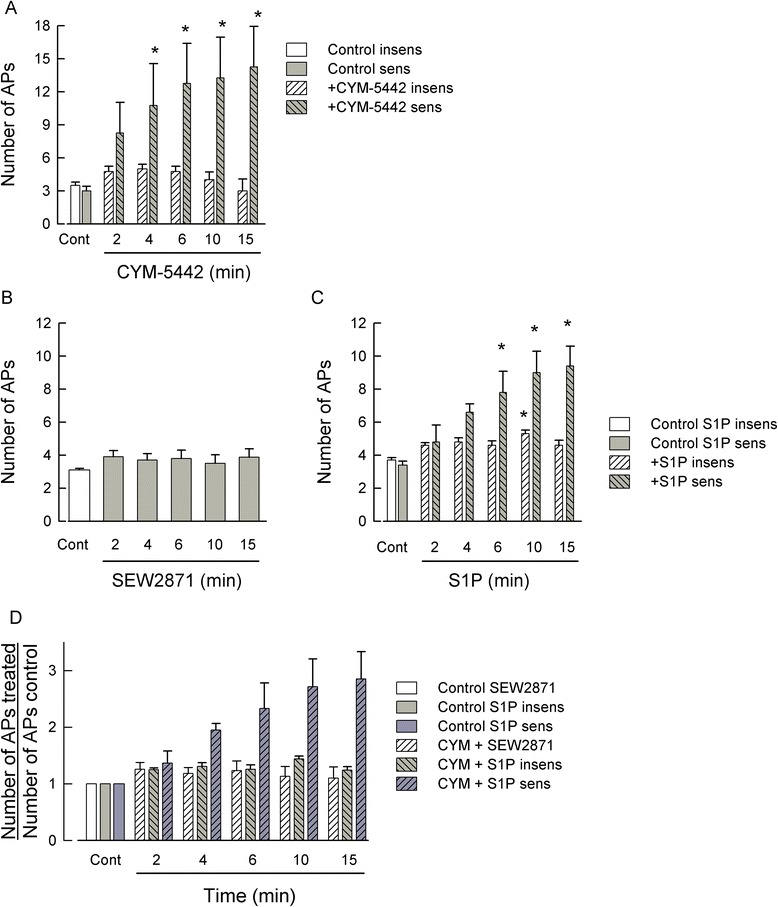
Acute CYM-5442 exposure increases neuronal excitability, but prolonged treatment blocks sensitization to SEW2871, but not to S1P. (**A**) summarizes the acute effects of 100 nM CYM-5442 on sensory neurons. In one group, CYM-5442 significantly increased the number of APs after only a 4-min exposure (*n* = 4), whereas the other group appeared to be insensitive to CYM-5442 (*n* = 4). (**B**) demonstrates that after a 1-h pretreatment with 100 nM CYM-5442, the S1PR_1_ selective agonist SEW2871 (100 nM) fails to increase the neuronal excitability (*n* = 10 control-10 min, *n* = 8 15 min). (**C**) shows that in a total of 15 sensory neurons, 10 were insensitive to 1 μM S1P although there was a small but significant increase in the number of APs measured only at the 10-min point. In contrast, five sensory neurons exhibited increased excitability in response to S1P. **(D)** demonstrates that after normalization of the number of APs to their respective control values, there was no difference in the average number of APs after exposure to 100 nM SEW2871 or in those neurons that appeared to be insensitive to 1 μM S1P. However, there was a significant increase in the number of APs in those sensory neurons that were sensitive to 1 μM S1P. For the 6-min point, the increase measured in the S1P-sensitive neurons was significant compared to all the SEW2871 time points, all the S1P-insensitive times except for the 10-min point, and the S1P-sensitive control. For the 10- and 15-min points, the increase measured in the S1P-sensitive neurons was significant compared to all the SEW2871 and S1P-insensitive time points, as well as the S1P-sensitive control and the 2-min point (*P* < 0.001 ANOVA Holm-Sidak all-pairs test). AP - action potential, Cont - control, S1P - sphingosine-1-phosphate.

CYM-5442 is a selective agonist for S1PR_1_, and treatment with this compound, like FTY720, leads to the internalization and ubiquitination S1PR_1_ [60,61]. Using a similar approach as described above for FTY720, acute treatment with 100 nM CYM-5442 produced a significant increase in the excitability of small-diameter sensory neurons (see Figure 9A). As with FTY720, in a total of eight neurons, four neurons were sensitized by CYM-5442 within a 4-min exposure; however, four neurons remained insensitive to CYM-5442 even after 15 min. To test the idea that a prolonged treatment with CYM-5442 could lead to the down-regulation of S1PR_1_, we found that after a 1-h pretreatment with 100 nM CYM-5442, exposure to the selective agonist for S1PR_1_, SEW2871 (100 nM), failed to augment the excitability (Figure 9B). For example, after a 10-min application of SEW2871, the average number of APs (3.5 ± 0.5, *n* = 10) was not different than that obtained under control conditions (3.1 ± 0.1, *n* = 10, *P* = 0.44, Kruskal-Wallis ANOVA). These results suggest that CYM-5442 led to the selective down-regulation of S1PR_1_. If that is the case, then after prolonged treatment with CYM-5442, exposure to S1P should reveal the contributions of other S1PRs, namely S1PR_3_, to the S1P-mediated sensitization. As shown in Figure 9C, in a total of 15 neurons, exposure to 1 μM S1P did not alter the number of evoked APs in 10 neurons, although there was a small but significant increase in the average number of evoked APs measured at 10 min (control 3.7 ± 0.2 APs *vs*. 10 min 5.3 ± 0.21 APs, *P* = 0.002 Kruskal-Wallis ANOVA). However, in five neurons, the number of APs was significantly increased to 9.4 ± 1.2 from a control value of 3.4 ± 0.3 APs (*P* < 0.001, RM ANOVA Holm-Sidak all-pairs test). To reduce the variability in those sensory neurons pretreated with CYM-5442 and then exposed to these different agonists, the number of APs obtained for the different treatments was normalized to their respective values obtained for the control condition. As summarized in Figure 9D, there was no difference in the normalized number of APs obtained after exposure to SEW2871 compared to those neurons that were insensitive to S1P whereas there was a significant increase in the number of evoked APs in those sensory neurons that were sensitive to S1P. Thus, these results indicate that approximately one third of these CYM-5442-treated sensory neurons exhibited an increased excitability after exposure to S1P and, based on the results described above, suggest that S1PR_3_ likely mediates this effect. In addition, these results are similar to our previous findings wherein treatment with siRNA targeted to S1PR_1_ blocked the sensitization produced by SEW2871, yet in one third of these neurons (three of nine total), S1P produced a significant, twofold increase in the excitability [15].

### S1P does not activate a membrane current in sensory neurons

**Figure 10:**
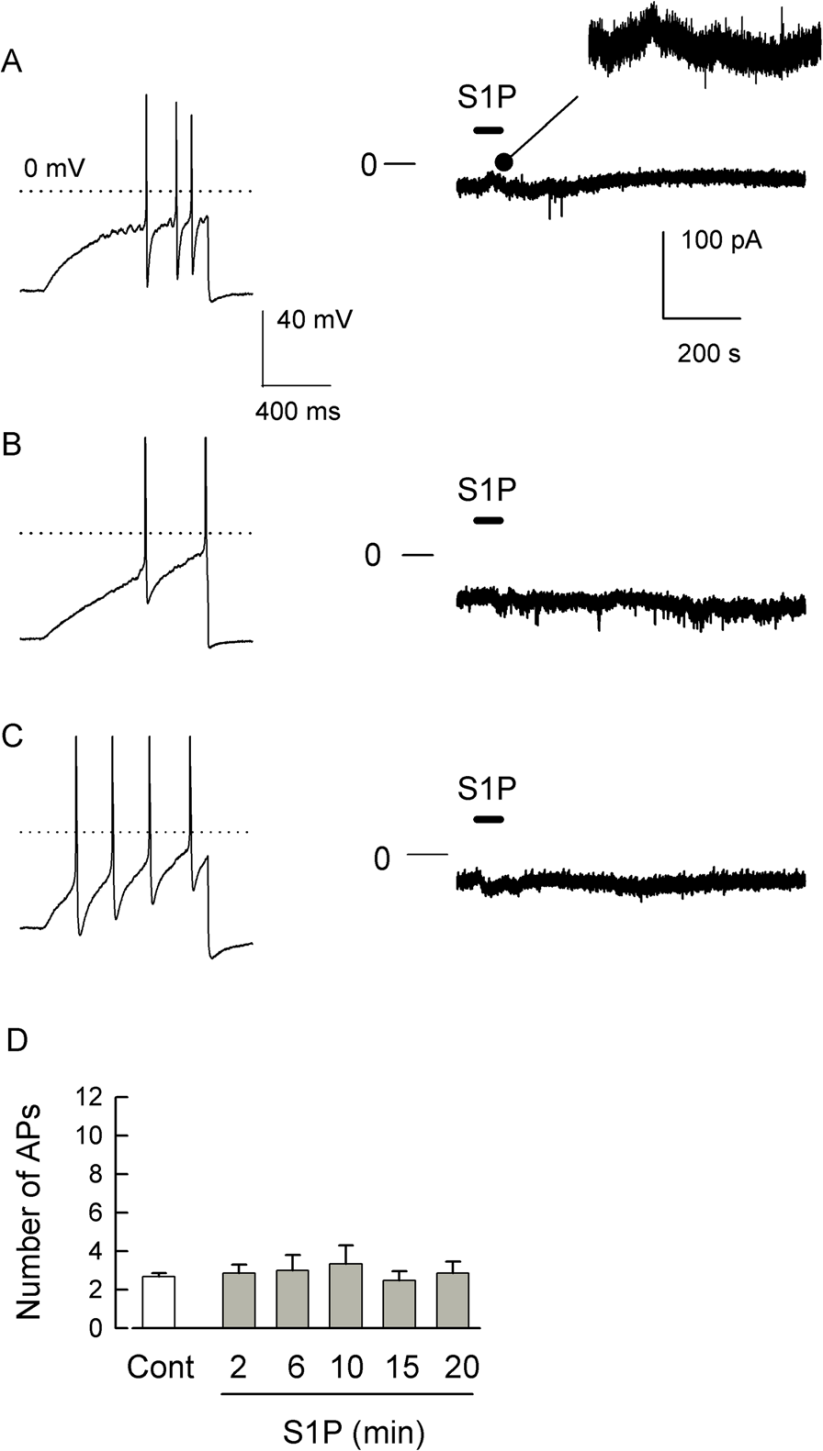
S1P does not evoke a change in membrane current in small-diameter sensory neurons. (**A**) Left panel shows a representative current-clamp recording where the ramp of depolarizing current evoked 3 APs in a small-diameter capsaicin-sensitive rat sensory neuron. The right panel shows the membrane current (holding voltage −60 mV) in response to a 60-s bath application of 1 μM S1P; the trace shows points at 20-ms intervals in order to reduce the dataset size; the inset represents an expanded portion of the current (35 to 235 s). (**B**) Left panel demonstrates a representative current-clamp recording where the ramp of depolarizing current evoked 2 APs in a small-diameter capsaicin-sensitive mouse sensory neuron. The right panel shows the membrane current (holding voltage −60 mV, 20-ms interval) in response to a 60-s bath application of 1 μM S1P. (**C**) Left panel shows that the ramp evokes 4 APs in this small-diameter capsaicin-sensitive mouse sensory neuron. The right panel shows the membrane current (holding voltage −60 mV, 20-ms interval) in response to a 60-s bath application of 100 μM S1P. In the left panels, the dotted line represents the 0 mV level; in the right panels, the line noted with 0 marks the zero-current level. (**D**) Medium- to large-diameter sensory neurons are not sensitized by S1P. Exposure of medium- to large-diameter sensory neurons isolated from rat DRG to 1 μM S1P by either bath superfusion (*n* = 7) or micro-injection of a stock into the bath (*n* = 8) does not alter the excitability. AP - action potential, Cont - control, S1P - sphingosine-1-phosphate.

A previous study [62] indicated that S1P, via S1PR_3_, was capable of directly mediating a membrane current conducted by chloride and is believed to result in the direct activation of nociceptive sensory neurons. As our work described above indicates that both S1PR_1_ and R_3_ mediate the sensitization of sensory neurons, the potential role of a S1PR_3_-induced current in regulating the excitability of sensory neurons was examined. As shown in a representative current-clamp recording obtained from a small-diameter (<25 μm) sensory neuron isolated from the rat DRG, a ramp of depolarizing current evoked 3 APs (left panel Figure 10A). However, in a voltage-clamp recording from this same neuron (holding potential of −60 mV, see the ‘Methods’ section for details), a 60-s exposure to 1 μM S1P via bath superfusion failed to produce a measureable change in membrane current (right panel). In a total of nine small-diameter capsaicin-sensitive sensory neurons (23.9 ± 0.6 μm), 1 μM S1P did not evoke any change in the existing membrane current. The studies performed by Camprubi-Robles *et al*. [62] used sensory neurons isolated from the mouse DRG. However, in small-diameter capsaicin-sensitive sensory neurons isolated from the mouse DRG, we found that 1 μM S1P did not evoke a change in membrane current (representative recording shown in Figure 10B, *n* = 7, average diameter 23.6 ± 0.7 μm). Furthermore, both 10 and 100 μM S1P failed to evoke any change in membrane current. In five mouse sensory neurons (average diameter 23.8 ± 0.4 μm, two capsaicin-sensitive, two capsaicin-insensitive, one lost before capsaicin application), 10 μM S1P was ineffective. In another three mouse sensory neurons (see representative recording in Figure 10C, average diameter 21.0 ± 1.0 μm, all three capsaicin-sensitive), 100 μM S1P evoked no change in the current. These results demonstrate that S1P, even at a high concentration, failed to elicit a change in membrane current in either rat or mouse small-diameter sensory neurons under our normal recording conditions. Although S1P can enhance the excitability of small-diameter sensory neurons, it appears to do so without evoking a direct change in membrane current. Based on this, the ability of S1P to enhance the excitability of medium- to large-diameter (>40 μm) sensory neurons isolated from rat DRG was determined. In these larger sensory neurons (average diameter 41.6 ± 0.4 μm), a 20-min exposure to 1 μM S1P did not increase the number of APs evoked by a ramp of depolarizing current (see Figure 10D, control 2.7 ± 0.2 APs, *n* = 15 *vs*. 20-min S1P 2.9 ± 0.6 APs, *n* = 13, *P* = 0.85 ANOVA on ranks). In addition, exposure to S1P did not alter the resting membrane potential in these neurons (data not shown, control −57.0 ± 0.9 mV *vs*. S1P 20 min −57.5 ± 1.6 mV, *P* = 0.87 ANOVA). S1P was applied by two different approaches, bath superfusion (*n* = 7) and micro-pipetting into the bath from a 100 μM stock solution (*n* = 8); neither of these delivery methods increased the number of evoked APs; the results obtained with these two methods were not statistically different, so they have been combined in Figure 10D. Taken together, these results suggest that small-, but not medium-large, diameter sensory neurons can be sensitized by S1P and that S1P cannot directly alter the membrane current in small-diameter sensory neurons isolated from either the rat or mouse DRG.

## Discussion

In this report, we demonstrate that S1P enhances the excitability of sensory neurons through the activation of S1PR_1_ and/or R_3_. A variety of approaches were used to isolate the contributions of specific receptors to the neuronal sensitization mediated by S1P. siRNAs targeted to individual S1PRs demonstrated that specific knockdown of the mRNA levels for S1PR_1_ and R_3_ were sufficient to prevent the sensitization produced by S1P. The results obtained with the specific agonist of S1PR_3_, CYM-5541, as well as pharmacological antagonists (W146, CAY10444, and the VPC compounds) are consistent with the idea that activation of S1PR_1_ and/or R_3_ augment excitability. Lastly, both FTY720 and CYM-5442 acutely increased the excitability of these sensory neurons. However, prolonged treatment with FTY720, which targets S1PR_1_, R_3_, R_4_, and R_5_, blocked the sensitization produced by either SEW2871 or S1P. In contrast, CYM-5442, which is selective for S1PR_1_, suppressed the effects of SEW2871 in all neurons, whereas the sensitizing actions of S1P still remained in approximately one third of the CYM-5442-treated sensory neurons. Therefore, these findings establish that the enhanced excitability produced by S1P results from the activation of S1PR_1_ and/or R_3_ but that R_2_, R_4_, and R_5_ are insufficient.

Our previous work indicated that S1PR_1_ played a prominent, although not exclusive, role in enhancing the excitability of small-diameter sensory neurons where treatment with siRNA targeted to S1PR_1_ completely blocked the SEW2871-induced sensitization, but in about one third of these siRNA treated-neurons, exposure to S1P was capable of producing significant increases in AP firing [15]. In a real-time single-cell qPCR study of the mRNA levels of the different S1PRs in isolated sensory neurons, we found that in small- (<25 μm, *n* = 18), medium- (25 to 40 μm, *n* = 17), and large-diameter (>50 μm, *n* = 17) neurons, S1PR_1_ was the highest expressing subtype in more than half (>10) of the total individual cells in each group [14]. In those neurons with S1PR_1_ as the highest expressor, five of the ten small- and five of the ten medium-diameter neurons expressed S1PR_3_ as the second highest subtype. In addition, there was a strong correlation between the expression of S1PR_1_ and R_3_ in both small- and medium-diameter sensory neurons (Pearson’s correlation coefficients of 0.89 and 0.92, respectively) [14]. Thus, after S1PR_1_, S1PR_3_ was the second highest expressor in approximately 50% of these identified neurons. These results are consistent with our previous siRNA studies examining the functional response of S1PR_1_ as well as those described above where the down-regulation of S1PR_1_ by CYM-5442 yields a group of neurons that were responsive to S1P, but not SEW2871. Based on the capacities of FTY720 and CYM-5442 to act as functional antagonists, these results suggest that after CYM-5442-induced down-regulation of S1PR_1_, S1PR_3_ remains capable of activation. The potential differences in cellular responses mediated by S1PR_1_ compared to S1PR_3_ may result from coupling to different G proteins and their respective downstream effectors. S1PR_1_ is believed to couple with only G_i/o_ whereas S1PR_3_ can couple with G_i/o_, G_q/11_, or G_12/13_, thus leading to the activation of a variety of effector systems; see reviews [5,63-65]. However, the specific roles of S1PR_1_ and R_3_ in the regulation of neuronal excitability remain to be defined and will be the focus of future investigations.

In addition, other studies support a role for S1P-S1PR_1_ in regulating the sensitivity of nociceptive sensory neurons. Opioid-induced hyperalgesia significantly decreased the latency of paw withdrawal to a thermal stimulus; this enhanced sensitivity was associated with a fourfold increase in the levels of S1P measured in the dorsal horn of the spinal cord [66]. Both the heightened sensitivity and the increase in S1P were blocked by pretreatment with either n-n-dimethylsphingosine or SK-I, inhibitors of sphingosine kinases. Injection of either S1P or SEW2871 into the rat or mouse paw produced thermal hyperalgesia ([13,10], respectively), which was blocked by treatment with W146, a selective antagonist for S1PR_1_ [13]. Localized perfusion of the L5 DRG with S1P increased the sensitivity of the rat’s paw to mechanical stimulation (by von Frey hairs) [67]. These authors also showed that localized injection of the inflammatory agent, zymosan, at the L5 DRG resulted in mechanical hypersensitivity of the hindpaw. However, a prior localized injection of siRNA targeted to S1PR_1_ at the L4/L5 DRG significantly reduced this hypersensitivity, suggesting that S1PR_1_ played a key role in the onset of this inflammatory-induced hypersensitivity [67].

An earlier study demonstrated that both intraperitonal and intrathecal delivery of FTY720 could reduce the nociceptive behaviors associated with either the inflammatory formalin model (number of flinches) or the neuropathic spared-nerve injury model (mechanical thresholds) [68]. Interestingly, effective doses of FTY720 did not have significant effects on the numbers of circulating white blood cells or lymphocytes, suggesting that the anti-nociceptive effects were not mediated by the immunosuppressive actions of FTY720. In contrast, the selective S1PR_1_ agonist, SEW2871, had no analgesic effect on the formalin-induced hypersensitivity.

Recently, it was shown that the intrathecal injection of SEW2871 produced a hypersensitivity (both allodynia and hyperalgesia) to mechanical stimulation of the rat’s hindpaw; this hypersensitivity was blocked by the S1PR_1_ selective antagonist, W146 [69]. Interestingly, the mechanical hypersensitivity resulting from the repeated injection of the chemotherapeutic agent, paclitaxel, was also blocked by W146 in a dose-dependent manner, suggesting that S1P-S1PR_1_ may play a role in the chemotherapy-induced peripheral neuropathy caused by paclitaxel. The peak of the increased sensitivity resulting from paclitaxel was associated with increased activity in the enzymes regulating the ceramide-sphingosine-S1P pathway, notably sphingosine kinase. Consistent with the idea that the paclitaxel-induced hypersensitivity was associated with S1P-S1PR_1_, prior intrathecal treatment with either FTY720 or CYM-5442 blocked, in a dose-dependent manner, the increased sensitivity caused by either SEW2871 or paclitaxel. Of significance, established paclitaxel-induced hypersensitivity could be reversed by exposure to either W146, FTY720, or CYM-5442, but not SEW2871. No effect on circulating white blood cells was observed. These results in combination with those results obtained by Coste *et al*. [68] strongly support the idea that antagonism rather than activation of S1PR_1_ is a key target in the suppression of this neuronal hypersensitivity. In future studies, it will be important to establish the signaling cascades that are activated by S1P-S1PR_1_ and determine the specific effectors that mediate the increased sensitivity as possible therapeutic targets.

A number of studies have established that the S1P-S1PR_1_ pathway plays a significant role in regulating the sensitivity of nociceptive sensory neurons through both cellular and behavioral approaches. However, in addition to our results described above, only one other study has explored the possible role of S1PR_3_ in regulating the sensitivity of sensory neurons. Camprubi-Robles *et al*. [62] demonstrated that S1P, presumably through activation of S1PR_3_, was capable of directly mediating a membrane current in nearly all sensory neurons isolated from the mouse DRG. Although neither the recordings of the reversal potential nor the concentration dependence were shown, application of 100 μM niflumic acid hastened the recovery phase of the S1P-induced current, suggesting that it was conducted by chloride. In current-clamp recordings, these authors report that 1 μM S1P, on average, depolarized the resting membrane potential from −54 to −36 mV with an increase in spontaneous AP firing. Our results demonstrate that FTY720 also depolarized the resting membrane potential by a similar amount (−54 to −39 mV); however, there was no enhancement of spontaneous activity, only AP firing evoked by the current ramp. We did observe a large depolarization in response to high concentrations of either CYM-5541 or SEW2871, and based on the suppressive effects of W146, this depolarization is thought to result from activation of S1PR_1_. In S1PR_3_−/− knockout mice, Camprubi-Robles *et al*. found that S1P depolarized the neuron by only approximately 5 mV; however, no membrane current recordings from the S1PR_3_−/− mice were shown. Using a fura-2-based assay, Camprubi-Robles *et al*. indicate that, in normal wildtype mice, approximately 60% of the neurons were responsive to 1 μM S1P and that niflumic acid reduced this to approximately 14%. However, in the S1PR_3_−/− mice, 40% of the neurons responded to S1P; if S1PR_3_ specifically mediates this response, it is curious why the knockout is not more similar to the actions of niflumic acid. Although the authors claim that this S1P-mediated current was exhibited by nearly all neurons, our experiments in both rat and mouse small-diameter sensory neurons failed to detect any measurable change in membrane current after exposure to even high concentrations of S1P (10 and 100 μM). In addition, S1P failed to augment the excitability in medium- to large-diameter rat sensory neurons. The basis for this difference remains an open question. One possibility could be differences in the culture media. In the Camprubi-Robles *et al*. study, sensory neurons were maintained in a synthetic serum-free medium supplemented with high levels of NGF (100 ng/ml) whereas, in our experiments, sensory neurons were maintained in an F-12 medium supplemented with 10% heat-inactivated horse serum and a lower concentration of NGF (30 ng/ml).

## Conclusions

Our results demonstrate that although S1PR_1_ plays a prominent role in enhancing the excitability of small-diameter sensory neurons, activation of S1PR_3_ can lead to the augmentation of current-evoked AP firing. Clearly, additional studies will be required to fully elucidate the mechanistic role of S1PR_3_ in regulating neuronal excitability and sensitivity to nociceptive stimulation. Important future work could establish whether there are significant functional interactions between S1PR_1_ and R_3_ or potential interplay between their downstream signaling pathways that mediate the sensitization of sensory neurons.

## Abbreviations

ANOVA: analysis of variance
APs: action potentials
Arbp: acidic ribosomal phosphoprotein P0
CHOs: Chinese hamster ovary cells
Cq: quantification cycle
dB: decibel
DRG: dorsal root ganglion
EC_50_: half-maximal effective concentration
EDTA: ethylenedinitrilo-tetraacetic acid
EGTA: ethylene glycol-bis(2-aminoethylether)-*N*,*N*,*N*′,*N*′-tetraacetic acid
GOI: gene of interest
HEPES: 4-(2-Hydroxyethyl)piperazine-1-ethanesulfonic acid
HPRT: hypoxanthine phosphoribosyltransferase 1
IC_50_: half-maximal inhibitory concentration
kHz: kilohertz
K_i_: inhibition constant
Meta: metafectene
MPL: 1-methyl-2-pyrrolidinone
ms: millisecond
mV: millivolt
MΩ: megaohm
NC: negative control
NGF: nerve growth factor
PGE_2_: prostaglandin E_2_
qPCR: real-time quantitative PCR
RM ANOVA: repeated measures ANOVA
S1P: sphingosine-1-phosphate
S1PR: sphingosine-1-phosphate receptor
siRNA: short-interfering RNA
SKI-II: sphingosine kinase inhibitor II
